# Cinnamaldehyde and Phenyl Ethyl Alcohol promote the entrapment of intermediate species of HEWL, as revealed by structural, kinetics and thermal stability studies

**DOI:** 10.1038/s41598-019-55082-1

**Published:** 2019-12-09

**Authors:** Zahra Seraj, Matthew R. Groves, Arefeh Seyedarabi

**Affiliations:** 1 Department of Biochemistry, Institute of Biochemistry and Biophysics, University of Tehran, Tehran, Iran; 2 Department of Drug design, University of Groningen, Groningen, The Netherlands

**Keywords:** Biochemistry, Biological techniques, Biophysics, Neuroscience, Plant sciences, Structural biology

## Abstract

Numerous efforts have been directed towards investigating the different stages leading to the fibrillation process in neurodegenerative diseases and finding the factors modulating it. In this study, using a wide range of molecular techniques as well as fibrillation kinetics coupled with differential scanning fluorimetry (DSF) and crystal structure determination of HEWL treated with cinnamaldehyde (Cin) and Phenyl ethyl alcohol (PEA) in their aroma form during fibrillation, we were able to identify the binding positions of Cin and PEA in HEWL. Additionally, crystal structures were used to suggest residues Thr43, Asn44, Arg45 and Arg68 as a plausible ‘hotspot’ promoting entrapment of intermediate species in the process of fibril formation in HEWL. We were also able to use DSF to show that Cin can significantly decrease the thermal stability of HEWL, promoting the formation of partially unfolded intermediate species. In conclusion, our data led us to emphasize that compounds in their ‘aroma form’ can influence the structure and stability of protein molecules and suggest reconsideration of HEWL as a model protein for fibrillation studies related to neurodegenerative diseases based on the initial structure of the proteins, whether globular (HEWL) or intrinsically disordered.

## Introduction

Aggregation of proteins is related to a wide range of neurodegenerative diseases such as Alzheimer’s and Parkinson’s diseases and is an intrinsic characteristic of most protein molecules^1–3^, as almost all proteins can aggregate under appropriate conditions. During aggregation, depending on the initial structure of the protein, unfolding of the protein may or may not be necessary, however, hydrophobic patches which are involved in aggregation are often exposed^4^. Therefore, fibrillation can occur when the rigid native structure of a protein is destabilized (as a common step in fibrillation), but the final product of the aggregation process could be either the amorphous aggregates, protofibrils/mature fibrils and/or soluble oligomers^5^. Since, the unfolded form of the protein in solution could help to increase aggregation, any compound which is able to stabilize the unfolded form of protein, directly or indirectly, could increase the aggregation/fibrillation process^4^. A variety of studies have used compounds from natural sources (such as polyphenols, flavenoids and polyamines) and tried to investigate their inhibitory effect on the fibrillation process. Some of the compounds which affect the fibrillation process are also found in their gaseous phase due to their small size and low vapour temperature, and hence produce an odour or aroma. In our previous study, we investigated the effect of three different aroma producing small molecules including Cinnamaldehyde (Cin), Phenyl ethyl alcohol (PEA), as two pleasant smelling compounds and active constituents of cinnamon and rose flower, respectively, and N,N,N,N′-Tetramethylethylenediamine (TEMED), as a foul smelling compound and representative of the smell of death, on the fibrillation process using HEWL as a model protein^6^. Based on the results achieved in our previous study it was clear that PEA and Cin were not able to stop the fibrillation process completely. They retained HEWL at oligomeric or protofibrillar stages and prevented mature fibril formation, while TEMED caused full inhibition. Therefore, in this study, our interest was on the intermediate species formed in the HEWL fibrillation process and hence we investigated the fibrillation kinetics of PEA and Cin on HEWL using a number of techniques and in particular differential scanning fluorimetry (DSF) and X-ray crystallography, to reveal the thermal stability and binding mode brought about by Cin and PEA, affecting the fibrillation mechanism in HEWL. We assumed that if we could assess HEWL at different incubation times during the fibrillation process in the presence of Cin and PEA in their aroma form, it would then help us to improve our knowledge about the exact mechanism of the fibrillation process, from the start to the end and even help find the intermediate stages in the fibrillation process. Additionally, findings from this study could help identify compounds which can stabilize the unfolded form of proteins, directly or indirectly, affecting the fibrillation process with different final aggregation products.

## Results

### Cinnamaldehyde can stop the process of fibril formation until 5 hours

**Figure 1:**
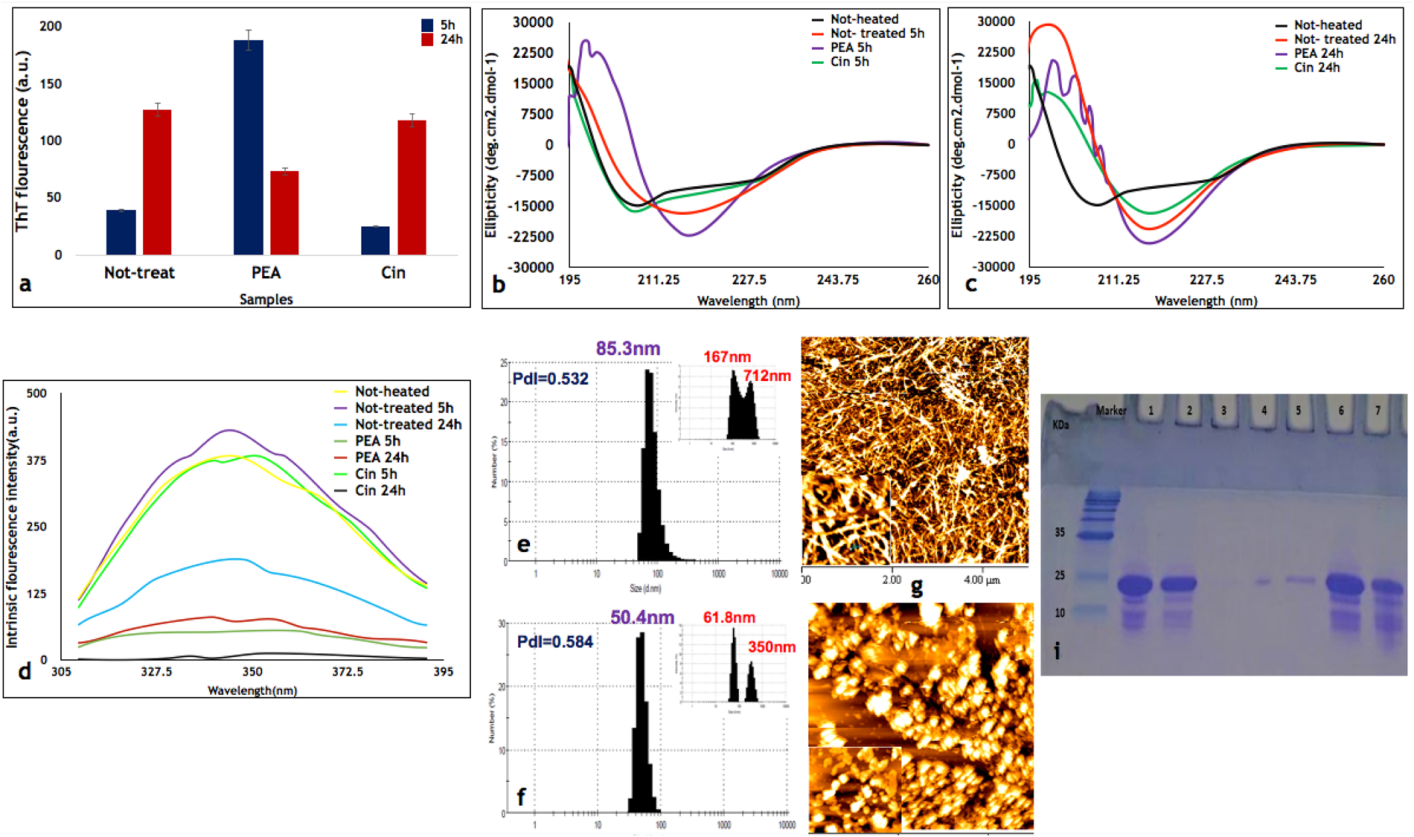
Characterization of HEWL samples treated with PEA and Cin after 5 and 24 h incubation. (**a)** ThT fluorescence intensities of HEWL with or without treatment with aroma of PEA and Cin after 5 and 24 h incubation. **(b, c)** Changes in the secondary structure of HEWL with or without treatment after 5 h and 24 h incubation under fibrillation conditions as monitored by CD. **(d)** Intrinsic fluorescence analyses of HEWL control and treated samples. The excitation wavelength was at 280 nm and the fluorescence emission intensity measured between 300 nm to 400 nm. **(e**–**h)** DLS and AFM of HEWL control and aroma treated samples: **(e, g)** PEA5h and **(f, h)** Cin5h. For DLS analysis all samples were diluted to 1 mg/ml. The intensity mode of each sample is also provided in the upper right corner of each panel. **(i)** The effect of aroma from PEA and Cin on HEWL fibrillation as assessed by SDS-PAGE. The wells contain the following: (Marker) Protein marker, (lane 1) Not-heated HEWL, (lane 2) Not-treated5h, (lane 3) Not-treated24h, (lanes 4, 5, 6 and 7) PEA5h, PEA24h, Cin5h and Cin24h, respectively.

For kinetics  studies, HEWL was prepared as described previously^6^ and then incubated at 54 °C in 50 mM glycine pH 2.2 for 4, 5, 6, 8 and 24 hours (h),  in the absence of aroma treatment, with the purpose of finding the starting point of fibril formation in Not-treated HEWL. CD spectroscopy and ThT fluorescence experiments confirmed fibril formation only after 4/5 h incubation and the secondary structure of HEWL changed from α-helix to β-sheet as incubation time progressed (Fig. S1). Following this preliminary fibrillation kinetics experiment, HEWL was incubated at 54 °C in 50 mM glycine pH 2.2 in the presence of aroma of Cin and PEA until 24 h. Based on the results achieved by CD and ThT fluorescence experiments (Fig. 1a and b), Cin could retain the alpha helical structure of HEWL until 5 h (Cin5h). However, PEA, which was previously shown^6^ to be able to slow down the process of fibrillation and retain the oligomeric form after 24 h incubation, was not able to conserve the native structure even until 5 h (PEA5h) and had transformed to the beta sheet structure (Fig. 1a–c). As the purpose of the fibrillation kinetics study was to determine whether Cin and PEA could be involved in the entrapment  of intermediate species (oligomeric/protofibrillar species) of HEWL, the incubation period of 5h and 24 h were selected for the rest of the analyses and comparisons in this study.

### Analyses of the effect of Cin and PEA on HEWL fibrillation after 5 hours incubation

The overall data obtained from ThT, CD, AFM, DLS and SDS-PAGE analyses for Cin5h revealed that in the presence of Cin, some intermediate sized species of HEWL was formed. The secondary structure analysis and ThT assays for Cin5h showed the same alpha helical content (Fig. 1b) and a low ThT fluorescence, respectively, compared to the Not-heated HEWL. Additionally, the migration of Cin5h on SDS-PAGE was also similar to Not-heated HEWL (in line with the CD and intrinsic fluorescence results), much more than Cin24h (Fig. 1i). DLS (Fig. 1f) and AFM (Fig. 1h) results showed molecules with larger diameter size and non-fibrillar shaped structures for Cin5h in comparison with Not-heated sample (the DLS and AFM of 24h incubated samples and their controls were reported previously^4^ and hence not shown again). On the other hand, continuing incubation for 24 h resulted in formation of fibrils with beta sheet structure in the presence of Cin (Fig. 1c), with the lowest intrinsic fluorescence intensity amongst the different HEWL samples (Fig. 1d), even lower than the Not-treated HEWL incubated for 24 h.

As for PEA, PEA5h (Fig. 1e and g) resulted in the formation of protofibrils with the diameter size of 85.3 nm (also in line with ThT fluorescence results), in comparison with mature fibrils formed in Not-treated HEWL incubated for 24 h with a diameter size of 193 nm^6^. SDS-PAGE analysis of PEA5h showed a reduced level of HEWL entry compared with PEA24h (Fig. 1i), in line with DLS results (Fig. 1e and^6^). Intrinsic fluorescence intensity results for PEA5h and PEA24h were almost the same, with PEA5h being slightly lower.

The results here emphasize that an intermediate species of HEWL is formed in the presence of Cin and PEA after 5 h, with Cin5h retaining the native secondary structure, while PEA5h had converted to the beta sheet structure.

### Thermal stability studies of HEWL in the presence of Cin and PEA in the aroma form

**Figure 2:**
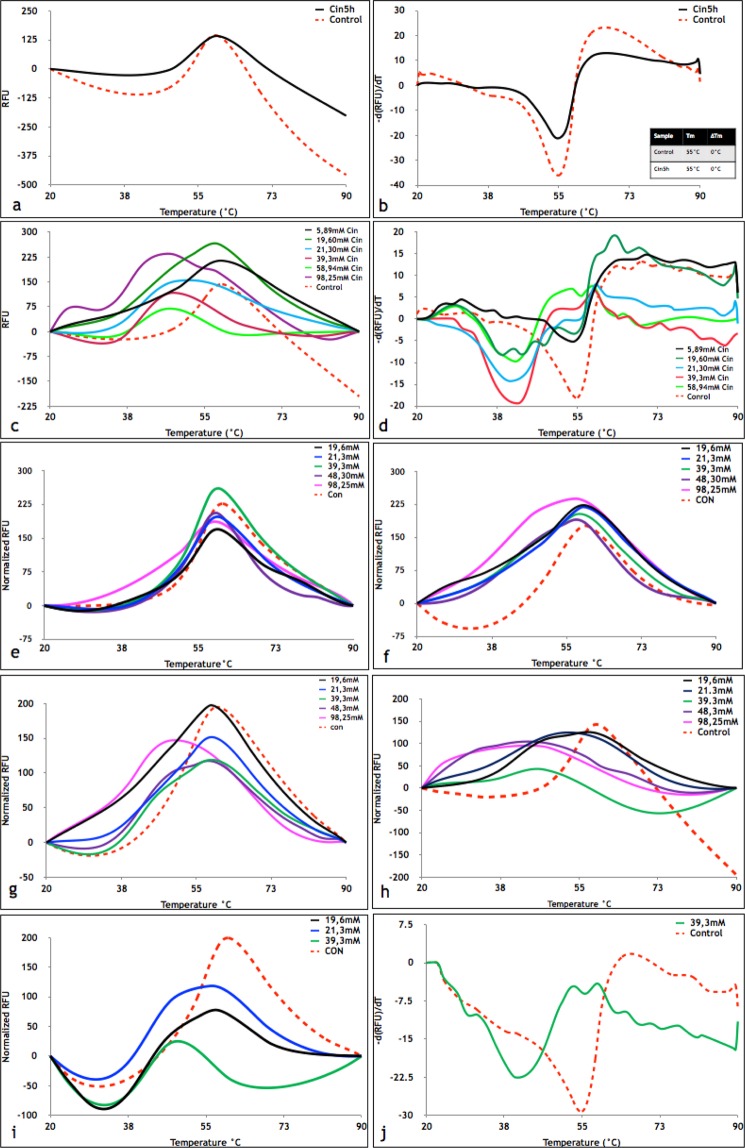
DSF of HEWL incubated with or without aroma of Cin. (**a**) Thermal melting profile of Not-heated HEWL (control) and HEWL treated with aroma of Cin for 5 h at 54 °C. **(b)** First derivative results of Not-heated HEWL (control) and HEWL treated with Cin for 5 h at 54 °C. **(c)** Thermal melting profile of Not-heated HEWL (control) and HEWL treated with different concentrations of Cin in solution at room temperature. **(d)** First derivative results of Not-heated HEWL (control) and HEWL treated with different concentrations of Cin in solution at room temperature. **(e–i**) Thermal melting profile of Not-heated HEWL (control) and HEWL treated with different concentrations of Cin for 2.5 2.5, 5, 7, 24 and 27 h, respectively, in solution. **(j)** First derivative results of Not-heated HEWL (control) and HEWL treated with 39.3 mM of Cin for 27 h. HEWL dissolved in 50 mM glycine buffer pH 2.2 was incubated with different concentration of Cin at room temperature for 2.5, 5, 7, 24 and 27 h, in solution.

Continuing our studies, we investigated the effect of Cin and PEA on the thermal stability of HEWL. HEWL was incubated under fibrillation conditions with the aroma of Cin and PEA and DSF was used to determine whether or not Cin or PEA resulted in shifts in the *T*_*m*_ value of HEWL (Figs. 2 and S2). In line with CD data with regards to the ability of Cin to maintain the secondary structure, DSF results confirmed that no changes were seen in the thermal stability of HEWL post incubation with the aroma of Cin for 5 h (Cin5h; Fig. 2a). On the other hand, as it was expected, thermal profiles of HEWL incubated for 24 h without any treatment (Not-treated 24h), in the presence of Cin (Cin24h) or in the presence of PEA (PEA24h), revealed a non-native structure, interpreted to be due to fibril formation (Fig. S2).

### Thermal stability studies of HEWL in the presence of Cin and PEA in solution

Since in this study, the aroma form of each of the small molecules were used, the exact effective concentration of each of these compounds were unknown. Therefore, as a next step, a range of different concentrations of Cin and PEA were calculated and used for incubation with HEWL dissolved in 50 mM glycine pH 2.2, for 24 h at room temperature. The concentration range was determined based on an assumption of equipartition of the aroma forming molecules similar to the vapour diffusion method, also used in protein crystallization (Table S1). As shown in Figure S3a and b, using different concentrations of PEA, no significant effect on thermal stability of HEWL was detected, which could be due to two different reasons. Firstly, it could have been because PEA did not have any effect on the thermal stability of HEWL. Secondly, the amount of PEA bound to the native state of HEWL was equal to the amount of PEA bound to the unfolded state^7^. However, when this experiment was repeated in the presence of different concentrations of Cin, a significant 13 °C decrease in thermal stability of HEWL was detected (Fig. 2c and d).

As shown in Figure 2b and d, it is clear that based on concentration and incubation time, the effect of Cin on thermal stability of HEWL ranged from no effect to reduction in the *T*_*m*_. Therefore, HEWL in its native form was further incubated with different concentrations of Cin for various durations of 2.5, 5, 7, 24 and 27 h (Fig. 2e–i). It was clear that only after 2.5 h incubation of HEWL with different concentrations of Cin, the thermal profile of HEWL treated with 48.3 mM and 98.25 mM Cin became multi-curved and a new peak at around 47 °C appeared (Fig. 2e), representative of the formation of a new population of HEWL in solution. Appearance of this peak at lower concentrations of Cin was observed after longer incubation periods and after 7 h incubation (Fig. 2g), most of the samples’ thermal profiles became multi-curved. Additionally, since there was no sign of a new population of HEWL in solution in the presence of low concentrations of Cin (19.6 mM, 21.3 mM and 39.3 mM) until 5 h, similar to Cin5h, it could be plausible to say that the concentration of Cin in the aroma form in the Cin5h sample could be in a range between (19.6 mM–39.3 mM) and hence the reason for not detecting a *Tm* reduction. After 24 or 27 h using 39.3 mM Cin (Fig. 2h,i), a single peak was observed and the *Tm* of HEWL was decreased by about 13.5 °C (Fig. 2j), in line with results shown in Figure 2d. Decrease in the thermal stability of HEWL in the presence of Cin shows that this small molecule has a greater affinity for the unfolded state of HEWL. As such, we see that after 24 h incubation of Cin with HEWL, the process of fibril formation can be facilitated in the presence of Cin.

### Structure determination of Not-heated HEWL and HEWL incubated in the presence of Cin and PEA aroma for 5 hours

Data collection was done at the XALOC beamline at the ALBA Synchrotron Source. After data reduction using iMOSFLM^8^, data scaling was done using Scala from CCP4 Software^9^, XDS^10^ and Xamuri, followed by molecular replacement using Phaser^11^. The model protein used was that of HEWL with PDB ID 1DPX. The best datasets were selected for refinement using refmac5 and the final structures were deposited in the protein data bank. Table 1 shows a summary of data collection and refinement statistics for structures of Not-heated HEWL (pH 2.2) as a control and HEWL incubated in the presence of Cin and PEA in their aroma form for 5 h, Cin5h and PEA5h, respectively.

**Table 1:** Data collection and refinement statistics.

Sample Name	PEA5h	Cin5h	pH 2.2
PDB ID	6AHH	6AHL	6AC2
Space group	P43212	P43212	P43212
a, b, c (Å)	a = 78.66, b = 78.66, c = 37.38	a = 78.42, b = 78.42, c = 37.25	a = 78.13, b = 78.13, c = 36.94
α, β, γ(°)	α = β = γ = 90	α = β = γ = 90	α = β = γ = 90
Resolution (Å)	37.38–2.1 (2.21–2.1)^a^	55.31–1.8 (1.9–1.8)^a^	55.25–1.23 (1.30–1.23)^a^
Total number of observations	72664 (9776)^a^	87510 (12540)^a^	317745 (43751)^a^
Total number unique	7282 (1037)^a^	11116 (1570)^a^	33801 (4811)^a^
Multiplicity	10 (9.4)^a^	7.9 (8)^a^	9.4 (9.1)^a^
Completeness (%)	100 (100)^a^	100 (99.9)^a^	99.9 (99.9)^a^
Rsym^b^ (Rmerge)	0.160 (0.37)^a^	0.194 (0.403)^a^	0.069 (0.423)^a^
Mean I/Sigma (I)	16.7 (9.0)^a^	7.2 (3.7)^a^	13.8 (4.5)^a^
Rpim^c^	0.076(0.185)^a^	0.073 (0.149)^a^	0.023 (0.147)^a^
Rmeas^d^	0.177(0.415)^a^	0.208 (0.430)^a^	0.073 (0.448)^a^
Resolution (Å)	2.1	1.8	1.23
rmsd bond length (Å)/angle ()	0.017/1.805	0.020/2.074	0.034/2.797
Mean B Factor (Å2)	10.821	19.475	16.292
R-factor/R-free (%)^e^	17.80/24.63	15.47/20.69	15.73/18.42

^a^The parameter values for higher resolution are given in parentheses.

^b^Rsym = ∑_hkl_ ∑_I_ |I_i_ −|/∑_*hkl*_ ∑*I*_*i*_, I^i^ is the intensity of the i^th^ observation, <I> is the mean intensity of the reflection and the summations extend over all unique reflections (hkl) and all equivalents (i), respectively.

^c^Rpim is a measure of the quality of the data after averaging the multiple measurements.

^d^Rmeas (also known as Rrim) is an improved version of the traditional Rmerge (Rsym) and measures how well the different observations agree.

^e^*R-factor* = ∑_*hkl*_ *|Fo_Fc|*/∑_*hkl*_ *Fo*, where Fo and Fc represent the observed and calculated structure factors, respectively. The R-factor is calculated using 95 % of the data included in refinement and R-free the 5 % excluded. The values presented in this Table come from SCALA^9^ and REFMAC^31^ from the CCP4 suite^28^.

### PEA binding sites in HEWL

**Figure 3:**
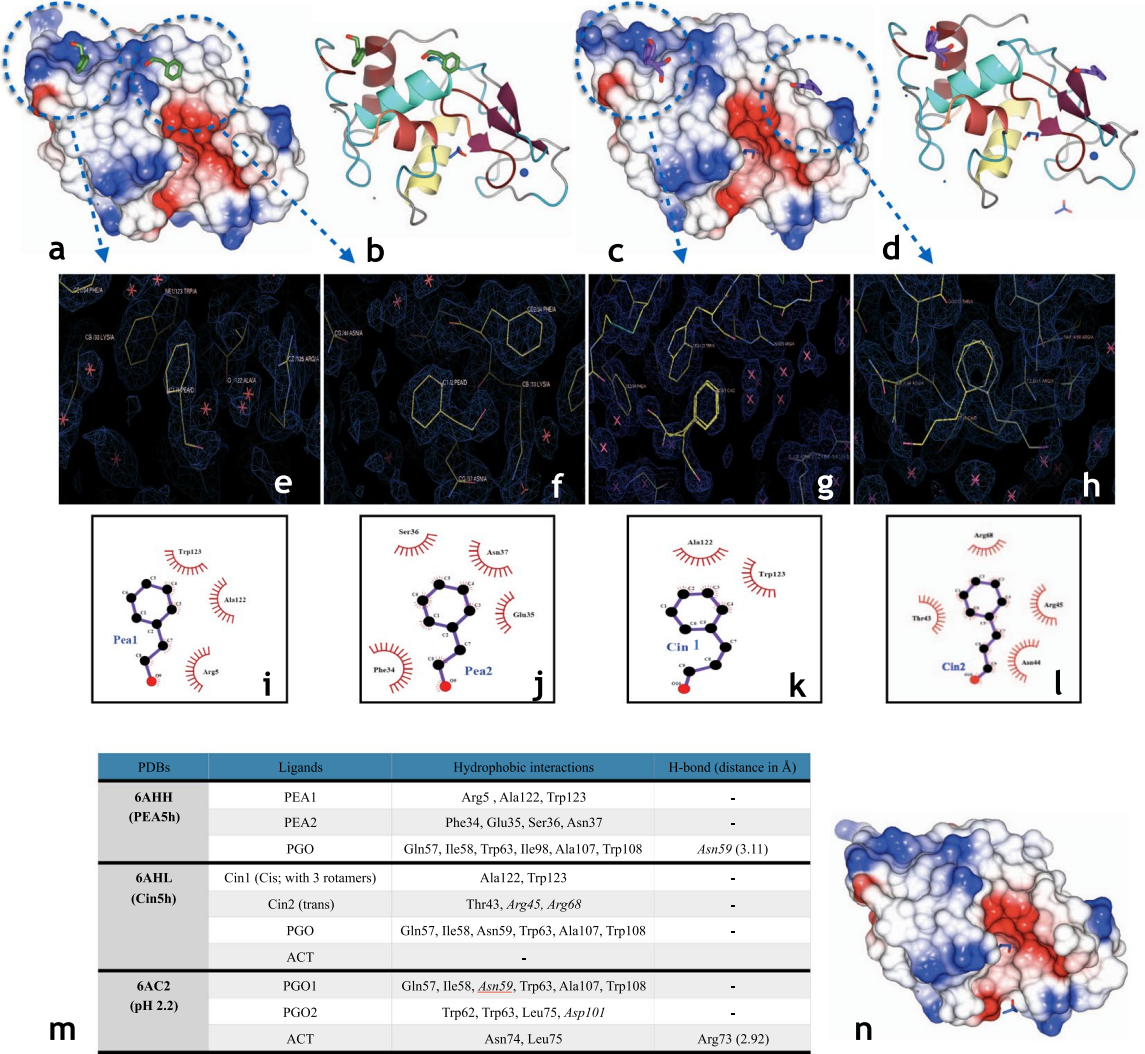
Overall graphical presentation view and ligand binding sites for PEA and Cin to HEWL. (**a, c)** Electrostatic potential representations of HEWL in complex with PEA and Cin, respectively, showed in their active site view. **(b, d)** The overall structures of HEWL-PEA and HEWL-Cin complexes, respectively, in the ribbon shape model. PEA and Cin molecules are coloured green and purple, respectively. Oxygen is coloured red. PGO and ACT are shown as sticks with carbon coloured blue and oxygen coloured red. **(e, f)** Electron density map of binding of PEA near Trp123 and Phe34 of HEWL, respectively, after 5 h incubation in the aroma form. **(g, h)** Electron density map of binding of Cin1 and Cin2 near Trp123 and Asn44 of HEWL, respectively, after 5 h incubation in the aroma form. The electron density σA-weighted map was contoured at 0.5 sigma and generated in Coot from CCP4 package version 2.10.7^30^. **(i, j)** Binding of PEA near Trp123 and Phe34 of HEWL, respectively, after five h incubation in the aroma form. **(k, l)** Binding of Cin1 and Cin2 near Trp123 and Asn44 of HEWL, respectively, after 5 h incubation in the aroma form was generated using the Ligplot + software, version 1.4.5^29^. **(m)** Ligand binding sites for PEA and Cin, cryoprotectants and acetate. The residues were involved in either hydrophobic interaction or hydrogen binding as revealed by Ligplot + software version 1.4.5^29^. Residues which have rotamers are shown in italics. **(n)** Electrostatic potential representations of HEWL dissolved in 50 mM glycine pH 2.2 shown in its active site view. The structural graphics of a, b, c, d and n were generated using CCP4MG version 2.10.7^28^.

Looking at the crystal structure of PEA5h (Fig. 3a and b), which was determined from a single crystal obtained after 30 days from the soluble fraction of HEWL incubated with PEA for 5 h through centrifugation, two PEA molecules were bound to the HEWL structure. One PEA molecule was placed near Trp123 (PEA1, Fig. 3e and  i), which could explain the quenching of the intrinsic fluorescence signal (as supported by Fig. 1d, where substantial quenching effect is seen for PEA5h compared to the Not-treated HEWL where there is no quenching), and the other PEA molecule was placed near Phe34 and Glu35 (PEA2, Fig. 3f and j). What is interesting in this structure is that the second PEA molecule is positioned close to the active site of HEWL (Fig. 3f and j). For further comparison, since no crystal structures were available for Cin24h and PEA24h, HEWL co-crystallized with Cin and PEA (Cin-co: PDB ID 6AGN and PEA-co: PDB ID 6AGR, respectively) were used. The structural comparisons showed no evidence of PEA binding near Asn44 in PEA5h, which was in contrary to the structure of PEA-co (Fig. S4a) as well as Cin binding near Asn44 in structures of Cin-co (Fig. S4c) and Cin5h (Fig. 3c–l). The absence of PEA near Asn44 in PEA5h is plausible as there is no evidence of rotamers for Arg68 (which normally has rotamers; Table S2) and the fact that Arg45 has a shift. These changes and the lack of free space due to the shift in Arg45 could also explain why there is no rotamer of Asn44. In contrary, in the case of PEA-co, only one PEA is attached to HEWL near Asn44 (Fig. S4a).

### Cin binding sites in HEWL

In the case of Cin, the structure of Cin5h showed the binding of two Cin molecules at two different positions (Fig. 3m), although with poor density (Fig. 3g and h), which could be explained by the results achieved from DSF experiments showing that Cin has more affinity for the unfolded state of HEWL. One Cin molecule was seen to bind near Trp123 (Cin1, Fig. 3g and k) and the other near Asn44 (Cin2, Fig. 3h and l) in the symmetry related space. Cin1 positioned near Trp123 appeared to have electron density for the aldehyde group in three different directions (Fig. 3g). There are two possibilities for the presence of these electron densities. One could be due to the presence of three rotamers of Cin1 existing with only the aldehyde group being flexible. The other possibility could be the stacking of three Cin molecules on top of each other with varying aldehyde group positioning. However, the latter possibility was more plausible as the refinement of three Cin1 molecules stacked upon each other was allowed by refmac5, while that of the three rotamers for the aldehyde group of a single Cin molecule was not. Besides, there is a report about the crystal structure of cinnamaldehyde^12^, showing that Cin was able to stack on top of itself to form crystals. Another observation with regards to the structure of Cin5h is that the *trans* form of Cin was changed to *cis*, in Cin1.

Similar to Cin5h, Cin1 in Cin-co binds near Trp123 (Fig. S4) and Cin2 binds near Asn44 (Fig. S4). However, there is no evidence of stacking of Cin1 near Trp123 in Cin-co, as there are no multiple electron densities for the aldehyde group even though the resolution of Cin-co is much higher at 1.08 Å compared to 1.80 Å for Cin5h. Although there is no structure for Cin24h but using the structural information for the presence of Cin1 near Trp123 in both Cin5h and Cin-co, it is possible to generalize it to Cin24h.

## Discussion

Data obtained from CD analysis for Cin5h and PEA5h revealed that while Cin5h was able to retain the alpha helical structure, presence of PEA could not prevent change in secondary structure of HEWL from alpha helical to beta sheet after 5 hours of incubation in the aroma form. ThT and intrinsic fluorescence data for Cin5h showed a low ThT fluorescence and a similar intrinsic fluorescence intensity to Not-heated HEWL. PEA5h, however, revealed the highest ThT fluorescence and a much lower intrinsic fluorescence intensity. Based on the information from the different binding modes of either Cin or PEA near Trp123 and the differences observed in the fluorescence emission intensity of HEWL, it is actually plausible to conclude that Trp123 is the main residue involved in hydrophobic quenching and hence the differences in the fluorescence emission intensity in HEWL. According to the DSF results achieved by incubation of the native state of HEWL with different concentrations of Cin in solution at room temperature, it was revealed that Cin decreased the thermal stability of HEWL (with max *ΔTm* ~ 13 °C using 39.3 mM of Cin). This reduction in thermal stability of HEWL could explain why in the presence of Cin the fibrillation process in HEWL was not stopped. It appeared that Cin destabilizes the folded state of HEWL in addition to the effect of the acidic pH of both the buffer and itself. Having said that, the acidic pH (2.2) was a constant factor in all samples including Not-treated5h, PEA5h and Cin5h. Therefore, it is plausible to say that the destabilization of HEWL was not only caused by the acidic pH but rather resulted due to the presence of the Cin molecule and its interaction with HEWL. Furthermore, incubation of HEWL with different concentrations of Cin resulted in various changes in *ΔTm*. Regarding the DSF results achieved from Cin5h, which was similar to Not-heated protein, its plausible to say that not enough Cin was available to affect the structure of HEWL or perhaps the affinity of Cin for both the native and unfolded states of HEWL was the same at the concentration used in Cin5h. However, as confirmed by DSF results, an increase in the concentration of Cin caused greater affinity and binding to the unfolded state resulting in decreased thermal stability of HEWL.

Frare *et al*. 2004, reported that the fragment comprising residues 41–60 in acidic solutions is involved in forming the oligomeric state of HEWL^13^. This sequence is related to the ß sub-domain of the HEWL structure and could be an important position for driving HEWL towards oligomer formation as the residues are surface exposed as shown in Figure 3. Hence, the presence of Cin at a position consisting of residues Thr43, Asn44, Arg45 and Arg68 can drive HEWL towards entrapping intermediate species. As mentioned before, based on DSF results, the presence of Cin destabilized HEWL and hence the poor density seen for the Cin molecule near Asn44 in the structure of Cin5h (Fig. 3). To confirm the binding position for Cin near Asn44 and further emphasize the link between the poor density and the DSF results, we can refer to the structure of HEWL co-crystallized with Cin (Cin-co : PDB ID  6AGN). In the Cin-co structure, Cin is seen to bind near Asn44 with good density and its presence further supported by the evidence that Asn44, which is seen to have rotamers in the structure of Not-treated HEWL (pH 2.2), has no rotamer in Cin-co as Cin occupies this space. Now coming back to the structure of Cin5h, when we reduce the electron density map rmsd level, we can see both the presence of Cin and the existence of rotamers for Ans44. This shows that Cin binding near Asn44  is transitory and Cin is bound  to a population of HEWL while the rest of the population has no Cin bound. It seems that during the first hours of incubation of HEWL with Cin, there are no Cin molecules near Asn44, similar to the structure of pH 2.2 (Fig. 3n), where Asn44 has two rotamers (A and B). This assumption is supported by DSF kinetic results using Cin, which showed that the second peak at around 47 °C (representing a new population of HEWL in solution), did not appear at the low Cin concentration in the range of 19.6 mM–39.3 mM, until 7 h (Fig. 3c). Therefore, with increasing incubation time (after 7 hours), Cin occupies this position and prevents existence of Asn44 rotamers (Fig. S5). Rotamer A of Asn44, which clashes with the site of Cin binding, would only exist if there is no Cin present, as otherwise it would be too close to the Cin molecule, in an impossible covalent bond forming distance. However, rotamer B of Asn44, which has clear density at 0.5 sigma and better, can be acceptably involved in hydrophobic interactions with Cin bound at this position. Besides, it should be added that Cin has a short half-life^14^, which also may explain the poor density seen (Fig. 3).

As we mentioned previously, Cin5h affects the fibrillation process by forming some kinds of intermediate species with the same secondary structure as that of native HEWL. DLS results showed that the polydispersity index of Cin5h had increased to 0.58 (Fig. 1i intensity mode) from 0.31 in Not-heated HEWL (data not shown). In other words, it seems that a large population of HEWL in Cin5h had a similar structure to the native HEWL, but the remaining population was composed of the intermediate state, which when incubated further for 24 h led to protofibril formation and changes in secondary structure from alpha helix to beta sheet.

Furthermore, self stacking of Cin is undesirable in fully stopping fibrillation as small molecules which have the ability to stack on themselves are not to be used as inhibitors of fibrillation, as they can instead cause the fibrillation process to be enhanced. Ideally small molecules which can be suitable candidates as inhibitors for protein aggregation should be able to interfere with the assembly process by binding to the hydrophobic region^15^. However, these small molecules should not be able to form beta-sheets by themselves^16^. Only then, these small molecules will be good candidates to be developed and screened as drugs against amyloidosis^15,16^. Therefore, looking at the results of Cin24h, the formation of the beta-sheet structures in amyloid fibrils, and the ability for Cin (Cin1) to stack on itself in the structure of Cin5h, leads us to conclude that Cin acts to promote entrapment of intermediate species in HEWL.

As for PEA5h, most of the data obtained for this sample, excluding the crystal structure, refers to the already changed alpha helical secondary structure of HEWL to the beta sheet and the formation of protofibrils (as another type of intermediate species), different to the oligomeric intermediate species seen in Cin5h (with alpha helical secondary structure). In the structure of PEA5h, there is no evidence of PEA bound near Asn44, which is suggested from the Cin5h crystal structure to be the residue involved in the entrapment of intermediate species of HEWL. As such, the crystal structure of PEA5h cannot be used as a true picture of the intermediate species entrapped by PEA, since also the crystal obtained for the structure determination of PEA5h was from a small soluble fraction of the sample (at a stage when the main population of HEWL was in the beta sheet confirmation i.e. the insoluble protofibrillar state). Centrifugation had allowed the separation of the soluble fraction from the insoluble fraction, giving rise to a single crystal after 30 days, resulting in the PEA5h structure, showing PEA bound to possible HEWL sites before the conversion of the alpha helical to the beta sheet conformation. In support of the identification of the hotspot for the entrapment of intermediate species of HEWL in this study, when looking at the PEA-co structure, it is clear that the one and only PEA molecule bound, is near Asn44, which under fibrillation conditions would turn the structure from alpha helical to beta sheet conformation, leading to the entrapment of protofibrils in HEWL.

## Conclusions

**Figure 4:**
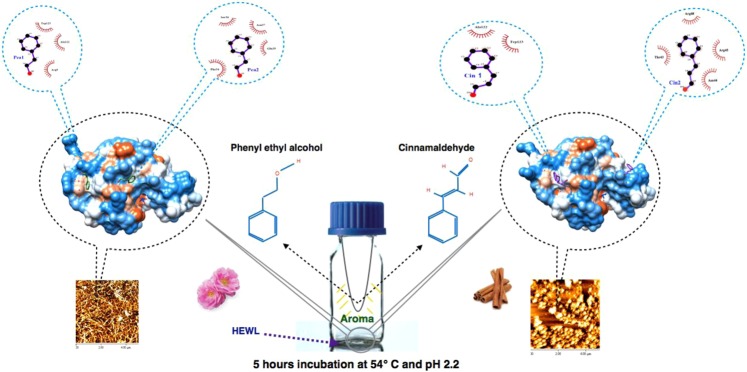
Influence of aroma form of PEA and Cin on fibril formation in HEWL.

In this study, we used structural analysis coupled with kinetics and thermal stability studies to unravel the effect of PEA and Cin in their aroma form on HEWL fibril formation. Previously, we reported that PEA and Cin (in their aroma form) were able to stop formation of mature fibrils in HEWL^6^. To take this further, in this study, we tried to find the binding sites of these small molecules to HEWL in their aroma form and search for hotspots necessary to entrap intermediate species. Listing the achievement from this study, the most interesting result was that the small molecules were able to affect fibril formation in their aroma form and bind to HEWL from their gaseous phase. Structural data coupled with a number of other experimental results in this study revealed the binding site of each of the small molecules in HEWL and suggested a hotspot comprising residues Thr43, Asn44, Arg45 and Arg68 to promote the entrapment of intermediate species of HEWL. DSF studies using different concentrations of Cin and PEA in solution showed that Cin could destabilize and facilitate partial unfolding of HEWL (Fig. 4). We did not see this destabilizing effect for PEA, perhaps because the amount of PEA bound to the native state of HEWL may be equal to the amount of PEA bound to the unfolded state^7^. In addition, DSF results revealed that Cin has more affinity for the unfolded state of HEWL than its native form, which raises a question whether or not the role of Cin and PEA for promoting the entrapment of HEWL intermediate species, could be generalized to the already unfolded proteins related to neurodegenerative diseases. In line with data achieved in this study, Ramshini *et al*. in 2015 reported that when cinnamon extract was used, some kind of small oligomeric species and not mature fibrils of HEWL was formed under fibrillation conditions^17^. On the other hand, Peterson *et al*. in 2009 reported that cinnamon extract inhibits tau aggregation (as an unfolded protein related to Alzheimer’s disease)^14^ and George *et al*. in 2013 reported the modulating role of cinnamaldehyde in Alzheimer’s disease^18^. Looking at some reports from different phenolic compounds (i.e. Apigenin^19–21^, Epigallocatechin‐3‐gallate^22,23^ and Quercetin^24^), which have the ability to stabilize unfolded structures or have direct interactions with the misfolded proteins, showed that they stabilize oligomeric species, resulting in an increase in the lag-time of the fibrillation process and a reduction in the amount of fibrillar structures or fibril growth. On the other hand, some of the mentioned compounds were reported to reduce fibrillation by re-directing the fibrillation process to an off-pathway and resulting in the production of non-toxic amorphous aggregates^25^. Therefore, these compounds were suggested as inhibitors for several aggregation-prone proteins, such as Aβ, α-synuclein and tau protein^25^. Regarding the difference in the initial structure of HEWL as a globular protein and the main proteins involved in neurodegenerative diseases, which are intrinsically disordered, it will be debatable whether a compound, which has the ability to destabilize a globular protein, making it prone to fibrillation, could have the same effect on an already unfolded protein. Based on this difference in the initial structures, we strongly suggest that the use of HEWL as a general model protein for neurodegenerative diseases be reconsidered.

## Materials and Methods

### Materials

Hen egg-white lysozyme or HEWL (catalogue number L6876), Phenyl ethyl alcohol or PEA (catalogue number W285803), Thioflavin T or ThT, Nile red, glycine (CAS No. 56-40-6), Sodium dodecyl sulphate (SDS) (Catalog no. 85,192-2), Trans-Cinnamaldehyde or Cin (lot MKBV8774V) and sodium acetate (CH3COONa) (lot 110H-072015) were all purchased from Sigma-Aldrich. Protein gel marker (PM1500) was purchased from SMOBiO. Mica for atomic force microscopy (ca. 92680) was purchased from PELCO. Acrylamide (UN-NO 2074), Amicon Pro-Affinity Concentration Kit Protein G (ACK5010PG) and N,N’methylendiacrylamid (EC.NO. 203-750-9) were purchased from Merck. NaCl (Lot 24091) was purchased from SERVA. 1,2 Propanediol was used from the Molecular dimension CryoProtX  kit. Crystal screen 2 reagent kits for crystal growth (CAT NO. HR2-112), 24 well crystallization VDX Plates with or without sealant (CAT NO. HR3-172 and HR3-142, respectively), OptiClear Plastic Cover Slides (CAT NO. HR8-074) and Siliconized Glass Cover Slides (CAT NO. HR3-239) were all purchased from Hampton Research. High vacuum grease was purchased from Girovac. For DSF experiments, Hen egg-white lysozyme or HEWL (Lot number P02C037) and Sypro Orange (catalogue number S6650) were purchased from Thermo Fisher. Glycine (Lot number AO359169) for DSF studies was purchased from ACROS. N,N,N,N′-Tetramethylethylenediamine or TEMED (CAS number 110-18-9) and PCR plates (catalogue number 82006-636) for DSF studies were purchased from VWR.

### HEWL sample solution preparation and incubation studies

Sample solutions of HEWL at 2 mg/ml in 50 mM glycine pH 2.2 was used in this study. PEA and Cin were used in their original purchased forms. The experimental set-up was such that 5 ml of 2 mg/ml HEWL was initially added to the bottom of an empty 100 ml Duran bottle; then 50 μl volume of aroma producing PEA or Cin were added to empty 50 ml falcon tubes with holes and placed inside the Duran bottle containing HEWL; the lids of the falcon tube and Duran bottle where sealed together. The Duran bottles containing HEWL as well as falcon tubes with aroma producing compounds inside, were incubated at 54 °C for 5 and 24 h (5 h and 24 h, respectively) in a shaker at 150 rpm for the process of aggregation/fibril formation to take place. The positive control HEWL solution, referred to as ‘Not-heated’ in this study, was also dissolved in 50 mM glycine pH 2.2 at 2 mg/ml but was not incubated. Another control HEWL solution referred to as ‘Not-treated’ also contained 2 mg/ml HEWL, which was incubated for 24 h but in the absence of either Cin or PEA^6^.

### Thioflavin T (ThT) fluorescence assay

HEWL samples incubated with or without aroma for 5 h or 24 h were diluted 50-fold with ThT solution (at 25 μM) and fluorescence intensities were recorded at 484 nm after excitation at 440 nm. Excitation and emission slit widths were both set at 5 nm. A sample of Not-heated HEWL was also used and ThT recorded as the control. The results were repeated and standard deviation bar calculated for the graph using multiple data.

### Intrinsic fluorescence intensity assay

Not-heated HEWL and treated or Not-treated HEWL were diluted 25 times with 50 mM glycine pH 2.2. The excitation wavelength was 280 nm and emission spectra were recorded between 300 and 400 nm. Excitation and emission slit widths were both set at 10 nm.

### Circular dichroism spectroscopy

Circular dichroism (CD) spectra of HEWL samples were recorded from 250 to 195 nm using an AVIV 215 spectrophotometer (Aviv Associates, Lakewood, NJ, USA). The sample preparations were the same as described before in our previous study^6^. Three scans of each sample were measured and averaged. The control buffer scans were run and subtracted from the sample spectra. The results were plotted as ellipticity (deg. cm^2^ dmol^−1^) versus wavelength (nm).

### Protein gel electrophoresis

Tris-glycine SDS polyacrylamide gel electrophoresis (SDS-PAGE) was used under reducing conditions to analyze the HEWL samples in this study. A pre-stained protein marker was used.

### Atomic force microscopy (AFM)

AFM scans were performed using a Veeco AFM instrument (Sharif University, Tehran, Iran). The sample preparation method was the same as we described before^6^.

### Dynamic light scattering (DLS)

Dynamic light scattering measurements were performed using the Malvern Zeta Sizer Nano ZS. The apparatus and parameters used were the same as we described in our previous study^6^.

### Differential scanning fluorimetry (DSF)

Stock solution (5000x) of the fluorescent dye Sypro Orange (Molecular Probes) was diluted with ultrapure water to a final concentration of 25x. The initial concentration of HEWL was 140 µM. Reaction mixtures were prepared by diluting HEWL in the presence or absence of desired concentrations of Cin and PEA and 5 µL of 25x Sypro Orange to yield a final reaction volume of 50 µl. The thermal stability was examined for all samples and 15 µM final concentration of HEWL was used. DSF experiments were performed in a BioRad CFX96 RT-PCR machine programmed for the temperature range 20–90 °C, and the heating rate 1 °C/0.5 min. All obtained curves were inspected manually to check their quality and the *T*_*m*_ values were determined using the first derivative curve. All experiments were performed in triplicates^26^.

### HEWL sample solution preparation and incubation studies for DSF

2 mg/ml (140 µM) HEWL solution was prepared in 50 mM glycine buffer pH 2.2. To prepare PEA24h, Cin24h, Cin5h and Not-treated24h, HEWL samples were incubated at 54 °C and 150 rpm for 5 and/or 24 h^5^, in the presence or absence of Cin and PEA. In addition, a second series of samples was prepared in which HEWL was incubated at room temperature for 24 hours in the presence or absence of different concentrations of PEA and Cin (Table S1). Additionally, kinetics study using different concentrations of Cin for a range of incubation time periods was done at room temperature.

### Crystallisation

Treated HEWL samples (at 2 mg/ml incubated with aroma form of Cin and PEA), were prepared as mentioned in our previous study^6^. For the purpose of crystallising, since the initial concentration of the incubated HEWL samples were 2 mg/ml, the incubated samples were concentrated before crystal screening using a concentrator with 10,000 Da MWCO. The final concentrations of HEWL from PEA5h and Cin5h were 7.5 and 7 mg/ml, respectively. Not-heated HEWL dissolved in 50 mM glycine pH 2.2, was also crystallised as the control. Conditions 1 (2 M NaCl and 10% PEG 6000) and 9 (2 M NaCl and 0.1 M sodium acetate pH 4.6) from Hampton Research Crystal Screen II were used for crystallisation of the HEWL samples. Crystals of Cin5h and PEA5h were obtained by optimizing conditions 1 and 9 and by varying the NaCl concentration from 1.2 to 2 M. Large crystals of HEWL treated with Cin5h were obtained after two days in the presence of 1.2 M, 1.4 M and 1.6 M NaCl. However, optimization did not help obtain crystals of PEA5h. Therefore, the PEA5h sample was further centrifuged at 14000 rpm (to remove any aggregated protein which hindered crystal growth) and used in crystallisation. Finally, after 30 days, only a single crystal was obtained for PEA5h from condition 1. Not-heated HEWL crystals were obtained from condition 9 after a 24 h period. The final cryoprotectant solutions were generally composed of the crystallisation conditions in which the crystals were obtained (with about 20 % increase in precipitant concentration) and the addition of 20 % (v/v) 1,2 Propanediol (PGO) as the cryoprotectant. All crystals were grown using the hanging drop method. The crystals were all flash frozen in liquid nitrogen in the presence of the cryoprotectant and used for data collection at the XALOC beamline, ALBA Synchrotron Source, Spain.

### Data collection and structure determination

Diffraction data were collected at the ALBA synchrotron source, the XALOC Beamline, at 100 K and at a wavelength of 0.9792 Å. The highest-resolution crystals diffracted to 1.08 Å. iMOSFLM^8^ was used for data reduction, while  Scala^9^, XDS^27^ and Xamuri (available at the XALOC beamline) for scaling and merging the intensities. The structures were determined by molecular replacement using Phaser^11^ and 1DPX was used as the search model. Refinement of the structures was performed using REFMAC version 5.8.0135^28^. Once the structures were solved (please refer to Table 1 for crystallographic data), Ligplot+^29^ and QtMG from the CCP4 software^28^ were used for a detailed structural analysis of the ligand binding site(s).

## Supplementary information

Supplementary information

## Data Availability

Data and material (where applicable) will be available upon request.
