# Supplementary material for: Cinnamaldehyde and Phenyl Ethyl Alcohol promote the entrapment of intermediate species of HEWL, as revealed by structural, kinetics and thermal stability studies
Source: Sci Rep. 2019 Dec 9;9:18615. doi: 10.1038/s41598-019-55082-1 (PMC6901479; doi:10.1038/s41598-019-55082-1)
Supplement: Supplementary file 1 — Supplementary information [file 41598_2019_55082_MOESM1_ESM.docx]

**Cinnamaldehyde and Phenyl Ethyl Alcohol promote the entrapment of intermediate species of HEWL, as revealed by structural, kinetics and thermal stability studies**

**Zahra Seraj^1,2^, Matthew R. Groves^2^ and Arefeh Seyedarabi^*1^**

^1^ Department of Biochemistry, Institute of Biochemistry and Biophysics, University of Tehran, Tehran, Iran

^2^ Department of Drug design, University of Groningen, Groningen, The Netherlands

*To whom correspondence should be addressed: Arefeh Seyedarabi

Department of Biochemistry, Institute of Biochemistry and Biophysics, University of Tehran, Tehran, Iran; Email: [a.seyedarabi@ut.ac.ir](mailto:a.seyedarabi@ut.ac.ir); Tel:+ (98) 21 66956974

**Supplementary information**

**Table S1.** Various concentration of small molecules used in this study using a vapour diffusion method.

| **Reduced amount from 50 µl (initial volume)** | **Dilution factor** | **Final concentration of Cin**  **(initial concentration divided by dilution factor)** | **Final concentration of PEA**  **(initial concentration divided by dilution factor)** |
| --- | --- | --- | --- |
| **3 µl** | 3µl/4000µl= 1333times | 7.86M/1333=5.89mM | 8.32M/1333=6.24mM |
| **10 µl** | 10µl/4000µl= 400times | 7.86M/400=19.60mM | 8.32M/400=20.8mM |
| **20 µl** | 20µl/4000µl= 200times | 7.86M/200=39.30mM | 8.32M/200=41.60mM |
| **30 µl** | 30µl/4000µl= 133times | 7.86M/133=58.94mM | 8.32M/133=62.415mM |
| **50 µl** | 50µl/4000µl= 80times | 7.86M/80=98.25mM | 8.32M/80=104mM |

**Table S2. Different rotamers of residues in HEWL in complex with small molecules and at different pH .**

| **Samples** | PEA-co | PEA5h | Cin-co | Cin5h | pH 2.2 |
| --- | --- | --- | --- | --- | --- |
| **PDB IDs**  Rotamers | 6AGR | 6AHH | 6AGN | 6AHL | 6AC2 |
| Arg14 | **Rotamer** | **NO-Rotamer** | **Rotamer** | **Rotamer** | **NO-Rotamer** |
| Asp18 | **NO-Rotamer** | **NO-Rotamer** | **NO-Rotamer** | **Rotamer** | **Rotamer** |
| Asn19 | **NO-Rotamer** | **NO-Rotamer** | **NO-Rotamer** | **NO-Rotamer** | **NO-Rotamer** |
| Asn44 | **NO-Rotamer** | **NO-Rotamer** | **NO-Rotamer** | **Rotamer** | **Rotamer** |
| Arg45 | **Rotamer** | **Rotamer** | **Rotamer** | **Rotamer** | **Rotamer** |
| Ile55 | **Rotamer** | **NO-Rotamer** | **Rotamer** | **NO-Rotamer** | **NO-Rotamer** |
| Asn59 | **NO-Rotamer** | **Rotamer** | **NO-Rotamer** | **NO-Rotamer** | **Rotamer** |
| Arg68 | **Rotamer** | **NO-Rotamer** | **Rotamer** | **Rotamer** | **Rotamer** |
| Asn77 | **NO-Rotamer** | **Rotamer** | **NO-Rotamer** | **NO-Rotamer** | **NO-Rotamer** |
| Ser85 | **Rotamer** | **Rotamer** | **Rotamer** | **Rotamer** | **Rotamer** |
| Lys97 | **Rotamer** | **NO-Rotamer** | **Rotamer** | **NO-Rotamer** | **Rotamer** |
| Asp101 | **NO-Rotamer** | **NO-Rotamer** | **NO-Rotamer** | **NO-Rotamer** | **Rotamer** |
| Val109 | **Rotamer** | **NO-Rotamer** | **Rotamer** | **NO-Rotamer** | **Rotamer** |
| Arg125 | **Rotamer** | **NO-Rotamer** | **NO-Rotamer** | **Rotamer** | **Rotamer** |
| Arg128 | **NO-Rotamer** | **NO-Rotamer** | **NO-Rotamer** | **Rotamer** | **Rotamer** |
| Leu129 | **Rotamer** | **NO-Rotamer** | **Rotamer** | **Rotamer** | **Rotamer** |


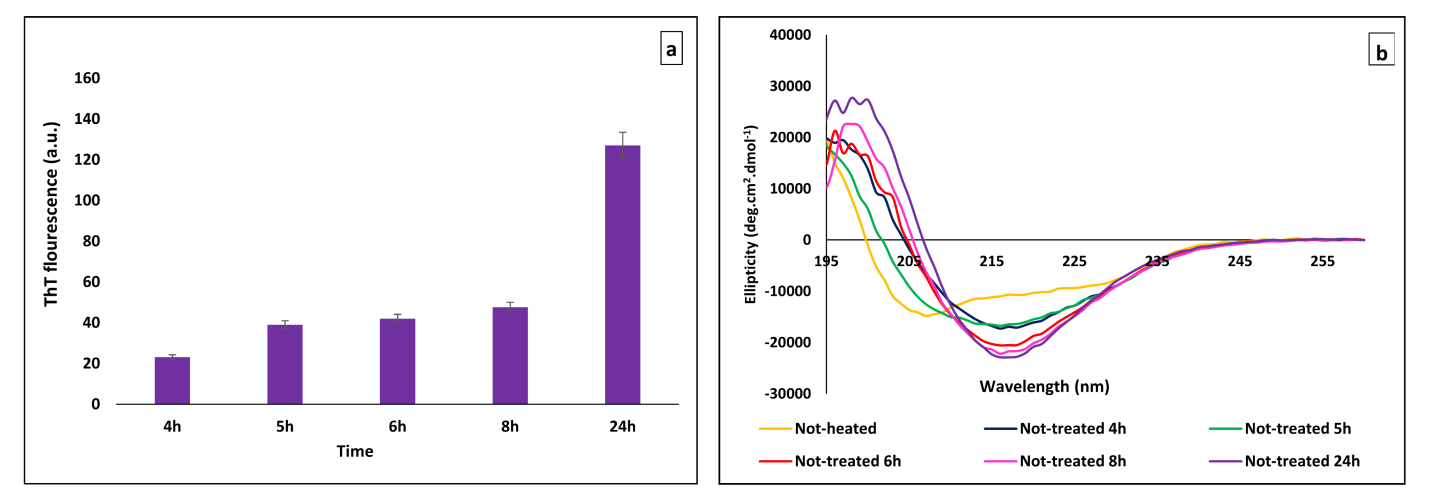


Figure S1. ThT and CD analyses of HEWL fibrillation process. (a) HEWL samples were incubated for 4, 5, 6, 8 and 24 hours in 50 mM glycine pH 2.2 at 54 °C and ThT emission measured at 484 nm. (b) Far-UV CD spectra of Not-heated HEWL and Not-treated HEWL after 4, 5, 6, 8 and 24h incubation.


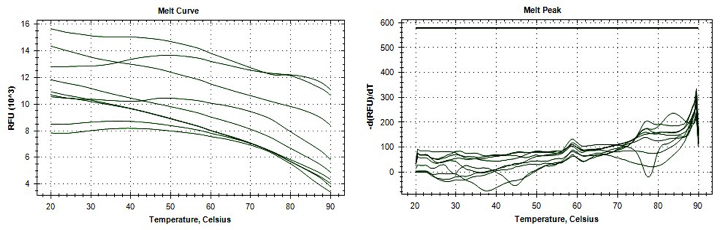


**Figure S2. DSF of HEWL incubated with or without aroma of PEA and Cin. (a)** Thermal melting profile of Not-treated HEWL (control) and HEWL treated with aroma of Cin and PEA for 24 hours at 54 °C. **(b)** First derivative results of Not-treated HEWL (control) and HEWL treated with aroma of Cin and PEA for 24 hours at 54 °C.


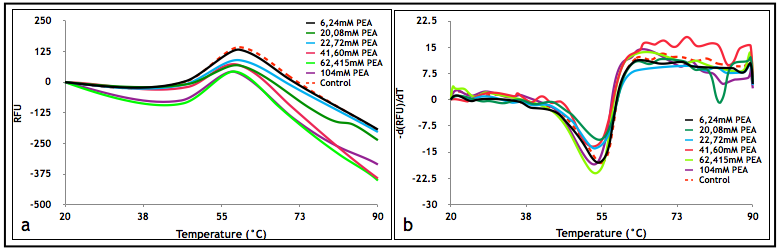


**Figure S3. DSF of HEWL incubated with or without aroma of PEA. (a)** Thermal melting profile of Not-heated HEWL (control) and HEWL treated with different concentrations of PEA in solution at room temperature. **(b)** First derivative results of Not-heated HEWL (control) and HEWL treated with different concentrations of PEA in solution at room temperature.


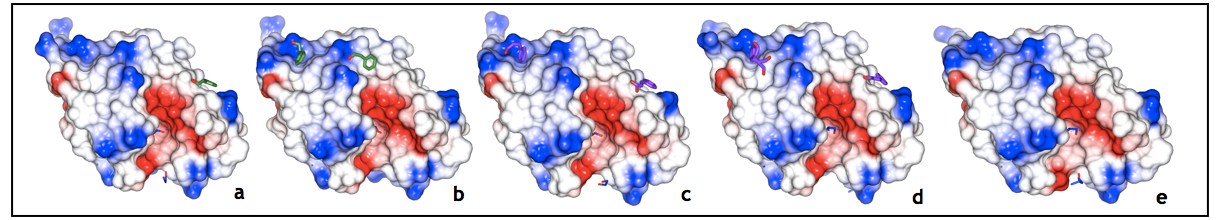


**Figure S4. Electrostatic potential representations of HEWL in complex with PEA, Cin.** **PEA and Cin** are coloured green and purple respectively. Oxygen atom are coloured red. PGO, EDO and ACT are shown as thin sticks with carbon coloured blue and oxygen coloured red. **(a)** PEA-co (PDB ID 6AGR), (**b)** PEA5h (PDB ID 6AHH), (**c)** Cin-co (PDB ID 6AGN), (**d)** Cin5h (PDB ID 6AHL), (**e)** HEWL in 50 mM glycine pH 2.2 (PDB ID 6AC2).


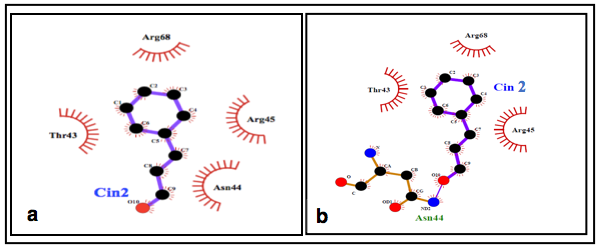


**Figure S5. Binding of Cin to HEWL as revealed by Ligplot analysis. (a)** Binding of Cin2 near Asn44 (rotamer B) of HEWL, after five hours incubation in the aroma form. **(b)** Binding of Cin2 near Asn44 (rotamer A) of HEWL, after five hours incubation in the aroma form. Figure was generated using the Ligplot+ software version 1.4.5.
